# Environmentally Specific Servant Leadership and Brand Citizenship Behavior: The Role of Green-Crafting Behavior and Employee-Perceived Meaningful Work

**DOI:** 10.3390/ejihpe13060083

**Published:** 2023-06-19

**Authors:** Ibrahim A. Elshaer, Alaa M. S. Azazz, Chokri Kooli, Ali Saleh Alshebami, Mohammad M. A. Zeina, Sameh Fayyad

**Affiliations:** 1 Department of Management, College of Business Administration, King Faisal University, Al-Ahsaa 380, Saudi Arabia; 2 Hotel Studies Department, Faculty of Tourism and Hotels, Suez Canal University, Ismailia 41522, Egypt; mohammed.zeina@tourism.suez.edu.eg (M.M.A.Z.); sameh.fayyad@tourism.suez.edu.eg (S.F.); 3 Department of Tourism and Hospitality, Arts College, King Faisal University, Al-Ahsaa 380, Saudi Arabia; aazazz@kfu.edu.sa; 4 Tourism Studies Department, Faculty of Tourism and Hotels, Suez Canal University, Ismailia 41522, Egypt; 5 The Telfer School of Management, The University of Ottawa, 75 Laurier Avenue East, Ottawa, ON K1N 6N5, Canada; ibm4chk@yahoo.fr; 6 Applied College, King Faisal University, Al-Ahsa 31982, Saudi Arabia; aalshebami@kfu.edu.sa; 7 Hotel Management Department, Faculty of Tourism and Hotels, October 6 University, Giza 12573, Egypt

**Keywords:** environmentally specific servant leadership, brand citizenship behavior, meaningful work, green crafting, job crafting, sustainable performance, hotel industry

## Abstract

Sustainability and environmental concerns have become increasingly important in the business world, with organizations seeking to integrate sustainable practices and enhance their brand citizenship behavior. Servant leadership that is focused on the environment is a type of leadership approach that gives prominence to preserving and promoting environmental sustainability. This study aims to examine the impact of environmentally specific servant leadership on brand citizenship behavior, with a focus on the mediating roles of green-crafting behavior and employee-identified meaningful work. Drawing on data from a survey of 319 employees working in hotels, this study conducted partial least square–structural equation modeling (PLS-SEM) to test a dual-moderated mediation model to explore the direct and indirect effects of environmentally specific servant leadership on brand citizenship behavior. The results of this study reveal that environmentally specific servant leadership has a significant and positive impact on green-crafting behavior and employee meaningful work. Moreover, green-crafting behavior and employee-perceived meaningful work both mediate the link between environmentally specific servant leadership and brand citizenship behavior. Specifically, green-crafting behavior acts as a mediator between environmentally specific servant leadership and employee-perceived meaningful work, while employee-perceived meaningful work mediates the link between green-crafting behavior and brand citizenship behavior. These findings have important implications for managers and organizations that seek to enhance their sustainability and brand citizenship behavior. Specifically, this study highlights the critical role of environmentally specific servant leadership (ESSL) in promoting green-crafting behavior and employee-perceived meaningful work, which in turn influence brand citizenship behavior. Therefore, organizations can improve their brand citizenship performance by developing ESSL behaviors and practices that foster green-crafting behavior and employee-perceived meaningful work.

## 1. Introduction

A strong brand is crucial for any service organization in the current, ever-changing, and unpredictable business environment [1]. Therefore, hotel companies, as one of the fastest growing and most aggressive competitive service sectors, are investing and performing better than ever to construct trustworthy brands [2] to distinguish their service and product offerings against competitors, boosting guests’ trust and satisfaction and diminishing perceived monetary, social, and safety hazards [3,4]. Unlike product brands, in which patrons’ perceptions of a brand are mainly derived from a product’s tangible attributes, guests’ perceptions of a service brand are heavily affected by the behavior of front-line staff [5]. In this context, scholars revealed that brand extra-role behaviors urge employees to go overhead with exceeding their standard in-role performance to fulfil the anticipations of the brand and patrons, thus developing a solid brand [6]. As a result, research on “brand citizenship behaviors” (BCB) has grown significantly in the past decade [7]. These behaviors drive employees not only to function as salespeople but also to display more empathy and willingness to satisfy patrons. They also make the connection between employees and the firm not a purely transactional connection [8]. This non-transactional relationship is definitely boosted by supportive leadership practices [9,10]. Nevertheless, there remains a limited understanding of how HRM could contribute to brand-building management; the factors that also drive employees toward BCBs, especially affective and cognitive functions, have yet to receive sufficient concentration [11,12].

Following this trend, scholars argued that servant leadership (SL) effectively supports followers to boost their level of BCBs [13]. The SL style is an emerging organizational phenomenon that enables a firm to portray and convey a positive corporate image as a distinctive brand [14] among current and potential employees and enables the positive influence of employee behavior outcomes, e.g., BCBs, via role modeling and constructive social exchanges [15]. However, SL research is still in its early stages; thus, further study is needed [13,16]. In the same vein, in the green context, the evidence chain has strengthened the positive link between “environmentally specific servant leadership” (ESSL) and employees’ discretionary behaviors, e.g., OCB, and more precisely “employee organizational citizenship behavior towards the environment” (OCBE) [17,18]. Yet, only a few empirical studies have analyzed how green leadership styles, including transformational leadership, responsible leadership, and ESSL as bottom-up leadership practices affect hotel employees’ non-green outcomes [19,20], such as BCBs and employee-perceived meaningful work. 

The SL style has been identified as distinguished among leadership theories because servant leaders are sincerely concerned for followers’ needs [21]. While the other leadership styles highlight performance and benefits for the firm, employee-perceived meaningful work might thus be improved depending on the SL mechanisms [22]. Similarly, ESSL practices make employees find meaning in green activities [23]. Then, experiencing meaningful work can encourage employees to feel they can benefit their organization by engaging in extra-role behaviors, e.g., BCBs, and thereby make an impact [24,25].

The “conservation of resources (COR) theory” [26] was built on the fundamental notion that employees tend to adopt a proactive resource gain strategy to amass additional resources and function beyond the minimum duties and expectations when they have abundant resources [27]. Accordingly, ESSL leaders’ practices, as a source of green-related resources, drive employees to proactively approach and accumulate green cognitive and motivational resources that enable employee green-crafting behavior with support from the perceived meaningful green work that motivates them to act beyond their roles [28], e.g., BCBs. Even with the importance of meaningful work and green-crafting behavior in green and non-green outcomes studies, to date, only some practical research has focused on such topics in this research area [29,30], especially in the hospitality industry. 

The present paper strives to contribute to the previous green literature by addressing some mentioned gaps above by using the COR theory, the basic theory relied upon in the present study, to test the link between ESSL and BCBs in the hospitality sector in a developing country while considering the mediating influences of green-crafting behavior (GCB) and employee-perceived meaningful work (MW) as well as the moderating effects of GCB on the two relationships in the proposed model. Thus, we aim to identify the role of ESSL practices in driving employees’ BCB behaviors and determine the impact that GCB and MW can have in this role for ESSL practices. Data were examined using structural equation modelling (SEM) with the smart-PLS method.

To achieve the purposes of this research, the next section provides a theoretical background about the interrelated relationships of the study main concepts. The next section discusses the research methods and materials adopted for data collection and analysis. The results of the collected data are then discussed. Discussion and implication are elaborated in the next section. Finally, conclusions, limitations, and future research directions are highlighted.

## 2. Theoretical Background

### 2.1. ESSL and Brand Citizenship Behaviour (BCB)

Greenleaf was the first to seek to introduce the SL approach among contemporary organizational theories in 1970 [31], and it has gained substantial attention in recent years, mainly in the hospitality sector [32,33], for its priorities that focus on serving followers’ requirements and needs first and foremost [34,35]. In line with a growing body of practical research that has proven the significant link between leadership and green performance level [36] and specifically confirmed the effectiveness of SL theory in anticipating sustainability activities [37], Robertson and Barling [38] coined the “environmentally specific servant leadership” (ESSL) concept as a manifestation of SL where the leadership activities are all concentrated on fostering green behaviors [23]. Thus, in contrast to other leaders who strongly emphasize performance and promote competitive and materialistic cultures where what one accomplishes is more significant than who one is, servant leaders consider that caring for employees should be intrinsic rather than just a means of enhancing business performance and financial success [39,40,41]. Hence, these leaders are more likely to promote environmentally harmonious cultures in which who one is outweighs what one achieves [22,42]. Similarly, ESSL leaders help and empower staff to contribute to the sustainability of the business and the community at large; consequently, their followers view them as role models who are committed to environmental goals and have pro-environmental principles [43]. These unique characteristics make ESSL leaders’ behaviors a vital resource that can push employees who work in the hospitality sector to be involved in discretionary and extra-role behaviors [44], e.g., brand citizenship behaviors (BCBs). 

“Customers׳ perceptions of a service brand depend highly on the behavior of frontline staff” [45]. Therefore, there is consensus among academics and industry experts that employees in service sectors are crucial to building a brand and to its success [46,47]. Thus, BCBs became an essential strategic aim and objective of hotel leaders and marketers in the hospitality sector environment. Based on the theory of OCB [48], BCB demonstrates the employee’s voluntary or discretionary behavior as benefiting and helping a particular brand [5,45]. According to Nyadzayo et al. [49], BCB includes two dimensions. The first is brand enthusiasm, in which employees strive to take on and engage in extra brand-developing endeavors such as involvement in marketing activities via sponsors or/and charity events [50], sharing guest opinions that reinforce a branding decision [51], and partaking in brand-focused events. The second is brand endorsement by tying the company brand to favorable word of mouth by recommending and suggesting the brand to family, friends, or others [52]. However, only prior investigations on the antecedents to OCB are extensive [53], and studies on BCBs are limited [54], and specifically, there is even less of this focus among studies about the hospitality sector. Regarding the linking between ESSL and BCB, less attention has been paid to servant leadership’s beneficial effects for arousing employee OCB in general and especially regarding BCB despite the rising body of studies connecting servant leadership to employee job performance [55]. In order to contribute to bridging this gap, the current study proposes the following hypothesis:

**Hypothesis** **1** **(H1).** *ESSL is positively linked with BCB.*

### 2.2. ESSL and Green-Crafting Behaviour (GCB) 

The “one-size-fits-all” viewpoint of conventional “job design theory” has been surpassed by workers’ proactive strategies to redesign their job themselves, conceptually known as “job crafting” [56]. Job crafting (JC) is defined as “the physical and cognitive changes individuals make in the task or relational boundaries of their work” [25]. “Job crafting” is a crucial matter specifically for hotel-frontline employees because they should adapt and self-design or redesign their roles and tasks to satisfy customers’ different and unpredictable demands and needs under the current changing conditions [57]. Therefore, this approach has become crucial in successful organizational transformation and increasing customer satisfaction in the hotel sector [58]. Nonetheless, there is a notable paucity of studies on employees’ job-crafting behaviors in the hotel business [59]. Similarly, with organizations going green, employees strive to possess green-related resources by proactively “crafting” their green tasks and roles to engage in green behaviors [23,60]. From this point, based on the viewpoint of JC as given in ref. [61], ref. [23] conceptualized the green-crafting concept as “changing resources and demands for pro-environmental activities to make these activities more meaningful”, and further adapted the [62] JC framework to construct a four-dimension framework of green-crafting behavior: “increasing green-related structural resources, increasing green-related social resources, and increasing green-related challenging demands, and decreasing hindering green task demands”. Based on the COR theory, employees working with ESSL leaders are able to accomplish these green-crafting dimensions in which they can strengthen their “green-related resources” by proactively striving to gain knowledge and skills related to green activities and participate in support and feedback for sustainable performance. Staff can also increase their “green-related challenges” by proactively additional green taking charge behaviors or participating in new green enterprises. Additionally, they can lessen the commonness of cognitive tasks or emotional exchanges related to green initiatives and activities, for example, by avoiding them as a coping style [18,62,63]. These arguments contribute to the hypothesis below:

**Hypothesis** **2** **(H2).** *ESSL is positively linked with GCB.*

### 2.3. ESSL and Employee-Perceived Meaningful Work (MW)

Employees frequently spend nearly two-thirds of the day at work; thus, if organizational leaders can consistently underline which the work tasks performed by the staff are considered meaningful, they will be more ready to make favorable changes and contributions to their firm by, for example, sharing their ideas, suggestions, and knowledge or by being whistle-blowers [64]. In organizational psychology disciplines, the idea of meaningful work (MW) has received widespread recognition [65]. MW is conceptually defined as “work experienced as particularly significant and holding more positive meaning for individuals” [66]. Work has become a significant area where individuals seek meaning in today’s society [67]. Studies have shown that many employees are willing to obtain much lower income in exchange for more MW [68]. Additionally, employees who experience a feeling of meaning in their work are more motivated and productive and experience greater well-being [69]; in contrast, a lack of MW is a direct cause of alienation, pressure, emotional tiredness, and boredom [70]. 

To construct a strong theoretical structure and identify the fundamental precursors of significant work, researchers turned to the “self-determination theory” (SDT). This theory is based on the belief that persons have specific innate psychological essentials and that satisfying such needs is crucial for individuals’ well-being, development [71], and feeling of meaningfulness [72]. In line with this, servant leaders retain a vital role in determining MW states by fulfilling employees’ innate psychological essentials, as stated in SDT [73]. MW is an essential component of the SL approach, in which Greenleaf [34] (who developed the SL style) expressed the following: “The work exists for the person as much as the person exists for the work. Put another way, the business exists to provide MW to the person as it exists to provide a product or service to the customer”, and Graham [74] argued that “servant leadership” has greater potential to be more “transforming” than “transformational” leadership because it arouses a powerful sense of meaningful work in all stakeholders. In a similar vein, ESSL leaders motivate, serve, and develop their followers to achieve environmental objectives and goals [43]; at the same time, employees find these green initiatives and activities more meaningful [23]. Hence, this discussion leads to the following third hypothesis:

**Hypothesis** **3** **(H3).** *ESSL is positively linked with MW.*

### 2.4. Green-Crafting Behavior (GCB) and Brand Citizenship Behavior (BCB)

Crafting jobs aids in enhancing employee–job relationships, thus improving employee well-being and having positive organizational effects (e.g., work engagement and augmented OCB) [75,76,77]. Some prior studies have examined the link between JC and OCB [57] and revealed that JC is significantly and positively associated with employees’ OCB [78] as well as its role in empowering employees and allowing them to amend the tasks and relational constraints of their job, thus assisting in their deeper involvement at work, which ultimately leads to an increased level of OCB [62], and expressly, Luu [30] indicated that green-crafting behavior has a substantial influence on OCB. Based on the fact that BCB is an element of OCB but is a brand-targeted factor and reaches far beyond the OCB purview through targeting external employee behaviors [79], e.g., recommending or suggesting brands to clients and/or providing clarifications to them about it and its advantages [80], we can thus propose the following:

**Hypothesis** **4** **(H4).** *GCB is positively linked with BCB.*

### 2.5. Employee-Perceived Meaningful Work (MW) and Brand Citizenship Behavior (BCB)

According to the “job characteristics theory” (JCT), employees must perceive their work to be meaningful before developing favorable attitudes and behaviors [81]. That is, employees must believe that their brand activities are meaningful [82] before enacting positive and discretionary brand-related attitudes and behaviors [83,84], i.e., BCB. Moreover, because MW’s augmented sentiments are a vital pathway to enhance employees’ well-being, MW is deemed an essential resource for job-related well-being in the framework of COR theory [85]. Moreover, some practical studies have revealed that experiencing the sense of MW positively correlates to OCB [69,86], albeit limited studies have investigated the relationship between MW and BCB. Nevertheless, according to these arguments, we can claim that the positive outcomes of the feeling of MW drive employees to show BCB. Hence, the below assumption is posited:

**Hypothesis** **5** **(H5).** *MW is positively linked with BCB.*

### 2.6. Green-Crafting Behavior (GCB) and Employee-Perceived Meaningful Work (MW)

JC is the operation by which employees redefine and reimagine their job designs in ways that are personally meaningful to them [25]. Employees who proactively “craft” their “job demands and job resources” experience more “meaningful work” both directly and indirectly as a result of their person–job fit being optimized [87]. Therefore, the positive psychology literature’s investigation on JC argues that JC is significantly connected to performance through MW [88]. Similarly, employees with stronger green values constantly redesign their job to fit their personal green values to experience great MW [89]. Hence, the hypothesis below is suggested:

**Hypothesis** **6** **(H6).** *GCB is positively linked with MW.*

According to what was previously mentioned, the literature shows a relationship between ESSL, MW, and GCB and between MW, GCB, and BCB. Thus, based on the integrated introductory evidence and the cited explanations of these proposed direct relationships in the study model, the following two hypotheses for the mediation links are proposed:

**Hypothesis** **7** **(H7).** *GCB mediates the link between ESSL and BCB.*

**Hypothesis** **8** **(H8).** *MW mediates the link between ESSL and BCB.*

### 2.7. Green-Crafting Behavior (GCB) as a Moderator

**Figure 1:**
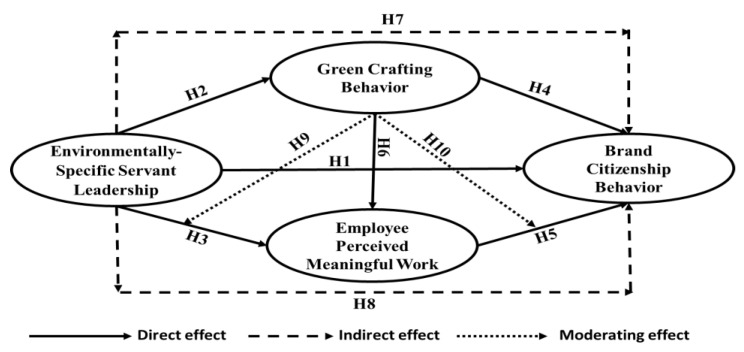
The research model.

In accordance with the “job crafting theory” of Berg et al. [88], employees can alter their jobs’ duties and social dynamics as well as how their work is perceived in general. These changes, e.g., adding favored tasks, creating stronger links with favorite co-workers, and sourcing better meaning to their jobs, facilitate employees to be more autonomous and skilled at work and experience more MW [78]. Accordingly, we argue that GCB is able to enhance the effect of ESSL leaders by improving the employee experience of MW and enhance the impact of MW by motivating employees to show BCBs. Consequently, the two following hypotheses are suggested by this study, as illustrated in Figure 1:

**Hypothesis** **9** **(H9).** *GCB moderates the impact of ESSL on MW.*

**Hypothesis** **10** **(H10).** *GCB moderates the impact of MW on BCB.*

## 3. Materials and Methods

### 3.1. Participants and Data Collection

Targeted employees of hotels in Sharm El-Sheikh, Egypt, were surveyed using a questionnaire to gather data. Sharm El-Sheikh city was chosen since it has so many highly rated five-star hotels. Workers who had a minimum of two years of experience were eligible to participate in the survey due to their adequate understanding in order to respond to the questionnaire. The data were collected from January to March 2022 using convenient sampling and drop-off and pick-up methods. Two phases of the survey were divided. Employees were oriented to supply the required data for ESSL, MW variables, and demographic data at the initial survey stage. The GCB and BCB variables questionnaire were completed by staff members at the same hotels one month after the first stage. A total of 600 questionnaires were disseminated among the two surveys. In response, 319 replies were taken into account, with an effective recovery rate of 53.2% after the unqualified forms were eliminated. Overall, 262 men (82.1%) and 57 women (17.9%) made up the study sample. The age range of the participants was primarily between 23 and 54.

### 3.2. Measures

All the variables’ questionnaire items were sourced from the literature and used. Using a 5-point Likert scale, all variables were evaluated. The ESSL variable was operationalized by the seven items proposed by Liden et al. [90]. The BCB was measured by the four items suggested by Helm et al. [12]. For the MW, three items were used from the study of Leiter [91]. Finally, the 21 items from the study of Tims et al. [62] were adopted to measure the GCB. The GCB scale items are divided into four dimensions, including five items for “increasing green-related structural resources”, five items for “increasing green-related social resources”, five items for “increasing green-related challenging demands”, and six items for “decreasing hindering green task demands”. The full study measures are presented in Appendix A. Furthermore, the survey questions were transcribed and edited to improve their clarity and comprehension. To ensure the survey’s validity, it was put to the test by eighteen people, including nine academics and nine industry professionals. Throughout these procedures, there was no modification to the survey’s content.

### 3.3. Data Analysis Methods

To test the proposed model, “structural equation modelling” (SEM) was carried out using “partial least squares” (PLS) by SmartPLS software V. 4.0. PLS is appropriate and applicable when the primary goal of the study expects one or more variables instead of validating a previously defined theoretical framework [92]. PLS-SEM is a convenient technique for the current study because it examines connections between the ESSL and BCB variables with the mediating roles of the GCB and MW between the ESSL and BCB and the moderating role of the GCB on ESSL towards MW and on the MW towards BCB. The PLS approach is additionally efficient across a broader range of sample sizes, is a more advanced model with fewer data restrictions, and is an efficacious tool [93]. Furthermore, compared to other statistical techniques, PLS-SEM enables the incorporation of more reflective items per variable. The PLS-SEM approach, according to Leguina [94], has two steps: “structural modelling and measurement modelling”. 

## 4. The Study Results

### 4.1. Outer Model

To examine data quality, the measurement model evaluates the “convergent validity” (CV) and “discriminant validity” (DV). The CV assesses the relationships between indicators by operating as the evaluation criteria “Cronbach’s alpha”, which must be more than 0.50 [95]; “composite reliability” (CR), which must exceed 0.60 [96]; “average variance extracted” (AVE), which should exceed 0.50 [93]; and “factor loading”, which is also preferably greater than 0.50 [97] as seen in Table 1 and Table 2. Additionally, DV asserts that the observed values must be discriminable when utilizing numerous approaches to measure other factors. In accordance with Fornell and Larcker [96], if the $\sqrt{AVE}$ of the factor is larger than the association between that factor and the other factors in the proposed model, the factor satisfies the statistical criteria for “discriminant validity” as seen in Table 3. Additionally, a number of researchers assessed the “heterotrait–monotrait” ratio of correlation (HTMT) to ascertain the “discriminant validity” in response to the various criticisms that were directed at “Fornell and Larcker’s criterion” [98] as seen in Table 4.

The CV values in Table 1 reveal that all of the suggested minimum and/or maximum levels were satisfied, demonstrating the suitability of the suggested outer model. Similarly, the scale’s $\sqrt{AVE}$ and HTMT values, as revealed in Table 2, both met the advised standards, demonstrating that its discriminant validity is sufficient (DV).

### 4.2. Hypotheses Evaluation 

The paper investigates collinearity issues utilizing the “variance inflation factor” (VIF) to ascertain if there exists any issue of collinearity among variables and to prevent the effect of the variables on the contribution of the proposed model. Based on Hair et al. [93], correcting multicollinearity is not necessary for VIF values below 5. The accuracy of the regression model in explaining the data was assessed through the use of the “coefficient of determination” (R^2^) and “Stone–Geisser’s” (Q^2^). In behavior studies, an R^2^ result of 0.20 is regarded as a high benchmark [93]. Likewise, Q^2^ scores reached the recommended point score of 0.0 [99]. Table 5 shows the VIF, R^2^, and Q^2^ findings.

Unlike “covariance-based SEM” (CBSEM), PLS does not provide multiple statistical measures for model validation, such as X^2^ and other model fit indicators [100]. To address this issue, “goodness of fit” (GoF) was introduced as an effective method for model validation [101]. According to Mital et al. [102] and Tenenhaus et al. [101], the following method can be used to calculate the GoF.

$$\mathrm{GoF}=\sqrt{{AVE}_{avy}\times {R^{2}}_{avy}}$$

Tenenhaus et al. [101] suggested that GoF values of 0.1, 0.25, and 0.36 indicate small, medium, and high GoF, respectively. The model proposed in this study has a GoF value of 0.619, which implies a significantly high GoF index. The model’s adequacy was further evaluated using the “standardized root mean square residual” (SRMR) by comparing variances in observed correlations. An SRMR value below 0.1 is indicative of a satisfactory model fit [103]. The SRMR value of the proposed model is 0.093, which suggests a good model fit.

After verifying the precision of both the outer and inner models, we proceeded to examine the proposed hypotheses of the study. To compute the regression weights (β), t-statistics, and the significance P level of direct, indirect, and moderating effects, a 5000 bootstrapping repetition was performed using Smart PLS4. Ten hypotheses were evaluated, comprising six direct hypotheses, two mediating hypotheses, and two moderating hypotheses, as outlined in Table 6.

**Figure 2:**
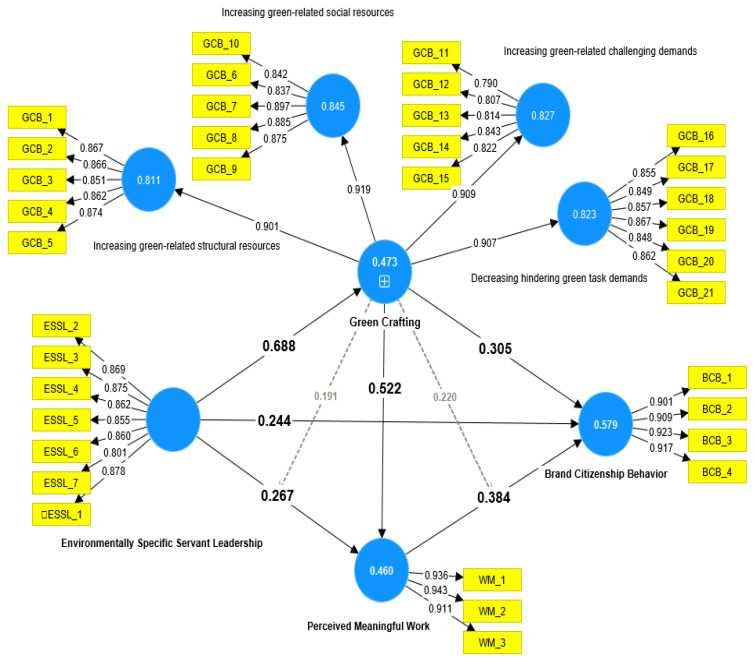
The study models.

Based on the calculations depicted in Table 6 and Figure 2, the ESSL had a significant and positive impact (*p* < 0.001) on BCB (β = 0.244, *t* = 3.570, *p* < 0.001), GCB (β = 0.688, *t* = 13.139, *p* < 0.001), and MW (β = 0.267, *t* = 2.597, *p* < 0.020), providing support for H1, H2, and H3. The findings also indicated that the GCB variable significantly and positively influenced BCB at β = 0.305, *t* = 3.732, and *p* < 0.001 and MW at β = 0.522, t = 5.986, and *p* < 0.001, confirming H4 and H6. Furthermore, MW positively affects BCB at β = 0.384, *t* = 7.184, and *p* < 0.001, supporting H5. Moreover, the variable of GCB and MW mediated the relationship between ESSL and BCB at β = 0.210, *t* = 3.495, and *p* < 0.001 and at β = 0.103, *t* = 2.256, *p* < 0.024, respectively, indicating that H7and H8 can be assumed.

**Figure 3:**
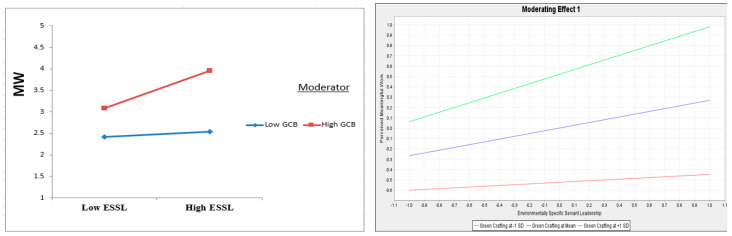
Interaction plot for GCB’s moderation influence on ESSL towards MW.

**Figure 4:**
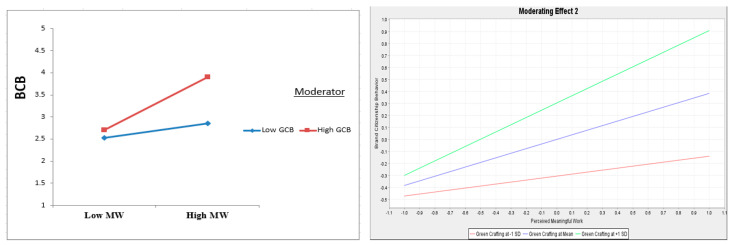
Interaction plot for GCB’s moderation influence on MW towards BCB.

The judgment of the moderating impacts revealed that the GCB has a significant impact on the correlation being assessed, as illustrated in Figure 3 and Figure 4. The smart-PLS analysis showed that GCB strengthened the significant positive influence of ESSL on MW (β = 0.191, *t* = 3.047, and *p* = 0.002), indicating support for H9. Similarly, MW strengthened the significant positive influence of MW on BCB (β = 0.220, *t* = 3.363, and *p* = 0.001), supporting H10.

## 5. Discussion and Implication 

The current study strives to respond to the contemporary research shift from corporate-level green outcomes to individual (employee)-level green behaviors in the hospitality sector [28,104], as employees are the critical performers that plan and implement green corporate policies [105]. Our study utilizes data gathered from the hospitality industry in Egypt, a developing country, to test the interrelationships of the ESSL and BCB through GCB and MW as mediators and GCB as moderator. Accordingly, the results of our practical study reached their goals and purposes by contributing to the green literature and theoretical development via the suggested model. The study’s results displayed that ESSL positively influences BCB (H1). Servant leaders, through their behaviors, make the organization a distinct and unique entity and enhance a positive employer brand image [14] that motivates employees to show increased BCB behaviors [16]. Furthermore, several empirical studies in the tourism and hospitality field have investigated servant leadership’s positive influences on followers’ attitudes and behaviors, including psychological empowerment [106], organizational commitment [107], work engagement [108], job satisfaction [109], employee innovative behavior [106,110], proactive client service performance [111], and organizational citizenship behavior (OCB) [55,112], thus effectively stimulating employees to enhance their BCB level [13]. Hence, ESSL follows the same pattern as servant leadership and may excel because it is more specific.

Furthermore, the study results found that ESSL positively affects GCB (H2). In general, servant leaders push their subordinates to maximize their abilities and promote their potential to the utmost, thus fostering the employees’ autonomy, which is needed for JC [90,113]. This is also supported by the empirical findings of Tuan’s study [23], which showed that ESSL was more positively correlated with employees’ GCB than green transformational leadership. 

On the other statistical path of the study model, in accordance with the argument that when servant leaders’ behavior signals employees that their work is valuable and significant, they may generate a more powerful feeling that it is meaningful and essential [114], our study proved that ESSL positively affected MW (H3). Here, Raub and Blunschi [115] also argued that CSR awareness, as one of the outcomes of ESSL practices, fosters the power of employees’ perceived meaningful work and in turn is positively related to job satisfaction and individual initiative and negatively associated with emotional exhaustion [116,117].

Concerning H4, the study findings proved that GCB positively affects BCB. Because JC enables employees to change their jobs to fit their talent and skills better [118], it leads to boosting psychological capital, job satisfaction, job involvement, and attachment and facilitates mobility into new functions and roles, thus motivating employees’ overall performance as well as discretionary behaviors (i.e., BCB) better than psychological empowerment and employee well-being [57,119]. Thus, the GCBs are helpful tools for leaders to motivate pro-environmental employees to exhibit voluntary behaviors, specifically BCBs.

In the same vein, the study results found that MW positively affects BCB (H5). According to the job characteristics theory (JCT), employees must believe their work is meaningful to develop desired attitudes and behaviors [120]. This means they must perceive their brand as meaningful to generate positive brand-related attitudes and behaviors, such as BCB behaviors [83]. 

Regarding the last direct hypothesis, the study result proved that GCB positively impacts MW (H6). The first scholars who investigated the relationship between GCB and MW also found support for our hypothesis [121]. These studies asserted that GCB is an essential factor and an antecedent to employees experiencing MW [25,122]. Scholars have indicated that JC enhances the person–job fit first, which then impacts the employee to perceive MW [121]. The current study, accordingly, argues that GCB positively influences the employee-perceived MW if the GCB aligns precisely with an employee’s green values [123].

One of our study’s primary targets was to test the mediating role of GCB and MW between ESSL and BCB. GCB, based on the study’s findings, successfully mediated the relationship between ESSL and BCB (H7). According to the conservation of resources (COR) theory [26], ESSL practices, “as a source of green-related resources”, can help shape GCBs among employees [23]. At the same time, employees who craft (redesign) their green duties and tasks may discover more meaningfulness in green practices and activities and thus strive to engage in volunteer and discretionary activities (e.g., BCBs) [113]. Similarly, MW succeeded in mediating the association between ESSL and BCB (H8). An organization’s ESSL practices act as a signal for pro-environmental employees, leading to heightened employee feelings of MW [89]. Thus, based on the supply–value–fit [124] and value–belief–norm [125] theories, we can argue that employees with pro-environmental attitudes, when they perceive meaningful work (MW), will feel a better fit and be more involved in positive outcomes (e.g., BCBs) for the organization [126], thus supporting GCB and mediating MW.

Finally, our “PLS-SEM” results reported the moderation effects of GCB on the connection between ESSL and MW (H9) and also on the relationship between MW and BCB (H10). Employee-perceived MW can fuel positive work behaviors by enabling employees to improve their prioritization and better concentrate on tasks [127]. According to the job characteristics model, employees engage in MW when job design or redesign (crafting) offers skill variation, task identity, task significance, autonomy, and feedback [120]. Thus, these psychological conditions will drive heightened satisfaction, performance [128], and other positive work outcomes (e.g., BCB). Consequently, GCB resulting from ESSL operationalization, besides its role as a mediator, could also strengthen the link between ESSL and MW and between MW and BCB as a moderator.

Finally, the fundamental managerial applications of the study can be drawn by recommending hotel leaders to work on improving BCB under the significant competitiveness, rapid growth, and great interest in environmental aspects at the level of organizations and individuals in this sector by adopting servant leadership strategies, especially those specific to the environment (ESSL), to facilitate GCB and strengthen the employee-perceived MW.

## 6. Conclusions, Limitations, and Future Research

Using the PLS-SEM approach, this paper highlights the impact of ESSL on BCB, with a focus on the mediating roles of GCB and employee-identified MW. The findings of this investigation indicate that the use of ESSL behavior considerably and positively influences both green CB and employee MW. Additionally, both green CB and employee-perceived MW mediate the association between ESSL and BCB. Specifically, GCB serves as a mediator between ESSL and employee-perceived MW, whereas employee-perceived MW acts as a mediator between green CB and BCB.

Future research could examine the effect of ESSL practices on employee well-being, employee retention and job satisfaction, as well as the connection between employee pro-environmental behavior and financial performance in general and green performance specifically. In addition, future examinations could discuss the impact of leader traits, such as individual green values, green passion, and the two dimensions of regularity theory, i.e., promotion focus and prevention focus, on adopting LSSE practices in the hotel sector. Additionally, since the study was restricted to the hotel sector in Egypt, it would be intriguing to see if the results can be extrapolated to other sectors such as manufacturing and service sectors in other different cultures by utilizing a different approach for data collection and/or analysis. Additionally, for the future research directions, comparative studies between regions and/or countries can be conducted. Finally, because the study was conducted using a cross-sectional survey method, it was difficult to draw conclusions about causality. Future analysis could operate longitudinal or experimental designs to demonstrate causal connections between LSSE practices, BCB, MW, and GCB.

## Figures and Tables

**Table 1:** Psychometric metrics.

	Estimates	(*a*)	(C.R.)	(AVE)
“Environmentally Specific Servant Leadership” (ESSL)		0.940	0.951	0.735
ESSL_2	0.878			
ESSL_2	0.869			
ESSL_3	0.875			
ESSL_4	0.862			
ESSL_5	0.855			
ESSL_6	0.860			
ESSL_7	0.801			
Green-crafting behavior (GCB)		0.966	0.969	0.598
“Increasing green-related structural resources” (STR)		0.915	0.936	0.746
Green-CB_1	0.867			
Green-CB_2	0.866			
Green-CB_3	0.851			
Green-CB_4	0.862			
Green-CB_5	0.874			
“Increasing green-related social resources” (SOR)		0.918	0.938	0.753
Green-CB_6	0.837			
Green-CB_7	0.897			
Green-CB_8	0.885			
Green-CB_9	0.875			
Green-CB_10	0.842			
“Increasing green-related challenging demands” (CD)		0.874	0.908	0.665
Green-CB_11	0.790			
Green-CB_12	0.807			
Green-CB_13	0.814			
Green-CB_14	0.843			
Green-CB_15	0.822			
“Decreasing hindering green task demands” (GTD)		0.927	0.943	0.734
Green-CB_16	0.855			
Green-CB_17	0.849			
Green-CB_18	0.857			
Green-CB_19	0.867			
Green-CB_20	0.848			
Green-CB_21	0.862			
“Employee-Perceived Meaningful Work” (MW)		0.922	0.951	0.865
MW_1	0.936			
MW_2	0.943			
MW_3	0.911			
“Brand Citizenship Behavior” (BCB)		0.933	0.952	0.832
BCB_1	0.901			
BCB_2	0.909			
BCB_5	0.923			
BCB_6	0.917			

**Table 2:** Factor cross-loadings.

	ESSL	STR	SOR	CD	GTD	MW	BCB
ESSL_1	0.878	0.661	0.619	0.593	0.566	0.500	0.625
ESSL_2	0.869	0.601	0.544	0.503	0.545	0.448	0.525
ESSL_3	0.875	0.614	0.522	0.450	0.379	0.420	0.521
ESSL_4	0.862	0.615	0.504	0.416	0.404	0.437	0.577
ESSL_5	0.855	0.592	0.503	0.447	0.393	0.429	0.554
ESSL_6	0.860	0.545	0.635	0.457	0.462	0.531	0.506
ESSL_7	0.801	0.500	0.575	0.629	0.634	0.680	0.602
GCB_1	0.626	0.867	0.742	0.736	0.707	0.415	0.505
GCB_2	0.605	0.866	0.741	0.677	0.740	0.418	0.408
GCB_3	0.562	0.851	0.626	0.618	0.487	0.382	0.430
GCB_4	0.595	0.862	0.704	0.575	0.580	0.413	0.440
GCB_5	0.573	0.874	0.698	0.636	0.558	0.372	0.421
GCB_6	0.633	0.778	0.837	0.612	0.644	0.467	0.362
GCB_7	0.593	0.709	0.897	0.680	0.670	0.502	0.449
GCB_8	0.512	0.638	0.885	0.631	0.628	0.465	0.399
GCB_9	0.531	0.671	0.875	0.731	0.663	0.516	0.513
GCB_10	0.569	0.739	0.842	0.720	0.657	0.487	0.537
GCB_11	0.458	0.651	0.667	0.790	0.513	0.417	0.471
GCB_12	0.494	0.625	0.691	0.807	0.681	0.506	0.577
GCB_13	0.501	0.541	0.648	0.814	0.607	0.443	0.370
GCB_14	0.545	0.644	0.651	0.843	0.728	0.565	0.470
GCB_15	0.405	0.610	0.516	0.822	0.709	0.451	0.418
GCB_16	0.432	0.612	0.666	0.711	0.855	0.579	0.545
GCB_17	0.512	0.592	0.644	0.717	0.849	0.520	0.519
GCB_18	0.472	0.599	0.583	0.645	0.857	0.538	0.486
GCB_19	0.482	0.627	0.617	0.619	0.867	0.533	0.448
GCB_20	0.476	0.558	0.602	0.658	0.848	0.465	0.402
GCB_21	0.568	0.696	0.742	0.738	0.862	0.587	0.443
MW_1	0.590	0.481	0.549	0.571	0.621	0.936	0.640
MW_2	0.515	0.415	0.503	0.533	0.582	0.943	0.597
MW_3	0.519	0.394	0.514	0.532	0.548	0.911	0.615
BCB_1	0.618	0.528	0.521	0.538	0.511	0.587	0.901
BCB_2	0.585	0.505	0.510	0.555	0.551	0.585	0.909
BCB_3	0.608	0.437	0.447	0.511	0.520	0.631	0.923
BCB_4	0.582	0.395	0.428	0.466	0.438	0.620	0.917

**Table 3:** Fornell–Larcker criterion matrix.

	BCB	GTD	ESSL	CD	SOR	STR	MW
Brand citizenship behavior	0.912						
Decreasing hindering green task demands	0.553	0.857					
Environmentally specific servant leadership	0.656	0.574	0.857				
Increasing green-related challenging demands	0.567	0.797	0.591	0.815			
Increasing green-related social resources	0.522	0.752	0.655	0.779	0.868		
Increasing green-related structural resources	0.511	0.718	0.687	0.754	0.816	0.864	
Perceived meaningful work	0.664	0.628	0.583	0.587	0.562	0.464	0.930

**Table 4:** HTMT matrix.

	BCB	GTD	ESSL	CD	SOR	STR	MW
Brand citizenship behavior							
Decreasing hindering green task demands	0.594						
Environmentally specific servant leadership	0.695	0.602					
Increasing green-related challenging demands	0.626	0.881	0.641				
Increasing green-related social resources	0.563	0.812	0.700	0.868			
Increasing green-related structural resources	0.552	0.770	0.740	0.839	0.886		
Perceived meaningful work	0.715	0.677	0.615	0.650	0.610	0.502	

HTMT: Heterotrait–monotrait matrix. For appropriate “discriminant validity”, all HTMT values need to be <0.90.

**Table 5:** VIF, R^2^, and Q^2^ results.

Variables	(VIF)	Variables	(VIF)	Variables	(VIF)	Variables	(VIF)	Variables	(VIF)
ESSL_1	3.891	GCB_1	2.915	GCB_8	3.368	GCB_15	2.254	MW_1	3.752
ESSL_2	3.730	GCB_2	2.841	GCB_9	2.996	GCB_16	2.894	MW_2	4.271
ESSL_3	3.747	GCB_3	2.533	GCB_10	2.293	GCB_17	2.861	MW_3	2.894
ESSL_4	3.926	GCB_4	3.112	GCB_11	2.318	GCB_18	2.827	BCB_1	3.292
ESSL_5	3.544	GCB_5	3.267	GCB_12	2.252	GCB_19	3.061	BCB_2	3.578
ESSL_6	3.106	GCB_6	2.319	GCB_13	2.333	GCB_20	2.934	BCB_3	4.348
ESSL_7	2.283	GCB_7	3.351	GCB_14	2.889	GCB_21	2.889	BCB_4	4.098
Brand Citizenship Behavior (BCB)					R2	0.579	Q2	0.464	
Green-Crafting behavior (GCB)					R2	0.473	Q2	0.281	
Employee-Perceived Meaningful Work (MW)					R2	0.460	Q2	0.380	

**Table 6:** Hypotheses testing (inner model results).

Hypotheses	β	*t*	*p*	Decision
Direct Paths				
H1—ESSL → BCB	0.244	3.570	0.000	“Supported”
H2—ESSL → GCB	0.688	13.139	0.000	“Supported”
H3—ESSL → MW	0.267	2.597	0.010	“Supported”
H4—GCB → BCB	0.305	3.732	0.000	“Supported”
H5—MW → BCB	0.384	7.184	0.000	“Supported”
H6—GCB → MW	0.522	5.986	0.000	“Supported”
Indirect Mediating Paths				
H7—ESSL → GCB → BCB	0.210	3.495	0.001	“Supported”
H8—ESSL → MW → BCB	0.103	2.256	0.024	“Supported”
Moderating Effects				
H9—ESSL * GCB → MW	0.191	3.047	0.002	“Supported”
H10—MW * GCB → BCB	0.220	3.363	0.001	“Supported”

## Data Availability

Data is available upon request from researchers who meet the eligibility criteria. Kindly contact the first author privately through e-mail.

## Appendix A

Study measurements

1. ESSL

− My manager can tell if something work-related is going wrong.
− My manager makes my career development a priority.
− I would seek help from my manager if I had a personal problem.
− My manager emphasizes the importance of giving back to the community.
− My manager puts my best interests ahead of his/her own.
− My manager gives me the freedom to handle difficult situations in the way that I feel is best.
− My manager would NOT compromise ethical principles in order to achieve success.

2. BCB

− I recommend the brand of my hotel to friends and family.
− I voluntarily take care of tasks that strengthen the brand of my hotel, even if those tasks are beyond my job responsibilities.
− To strengthen the brand, I voluntarily fulfill tasks which I think are important for the brand of my hotel much better than I am required to.
− I voluntarily engage in strengthening the brand of my hotel above and beyond what is expected of me, even if I am not directly rewarded by management for doing so.

3. MW

− This job provides me with opportunities to do work which 1 feel is important.
− My job provides me with successes which make me feel great.
− If 1 were choosing a new career, it would share many of the features of this job.

4. GCB

▪ Increasing green-related structural resources
  − I try to develop my environmental capabilities.2
  − I try to develop myself in terms of environmental knowledge and skills.
  − I try to learn new things about environmental improvement.
  − I make sure that I use my environmental capacities to the fullest.
  − I decide on my own how I do things about environmental improvement.
▪ Increasing green-related social resources
  − I ask my supervisor to coach about environmental knowledge and skills.
  − I ask whether my supervisor is satisfied with my green activities.
  − I look to my supervisor for inspiration about green activities.
  − I ask others for feedback on my green performance.
  − I ask colleagues for advice on my green activities.
▪ Increasing green-related challenging demands
  − When an interesting green project comes along, I offer myself proactively as a project co-worker.
  − If there are new environmental developments, I am one of the first to learn about them and try them out.
  − When there is not much green work to do, I see it as a chance to start new green projects.
  − I regularly take on extra green tasks even though I do not receive extra salary for them.
  − I try to make my green tasks more challenging by examining the underlying relationships between aspects of my green tasks.
▪ Decreasing hindering green task demands
  − I make sure that my green activities are mentally less intense.
  − I try to ensure that my green activities are emotionally less intense.
  − I manage my green activities so that I try to minimize contact with people whose problems affect me emotionally.
  − I organize my green activities so as to minimize contact with people whose expectations are unrealistic.
  − I try to ensure that I do not have to make many difficult decisions on green tasks.
  − I organize my green activities in such a way to make sure that I do not have to concentrate for too long a period at once.
